# Skeletal Muscle Depletion and Markers for Cancer Cachexia Are Strong Prognostic Factors in Epithelial Ovarian Cancer

**DOI:** 10.1371/journal.pone.0140403

**Published:** 2015-10-12

**Authors:** Stefanie Aust, Thomas Knogler, Dietmar Pils, Eva Obermayr, Alexander Reinthaller, Lisa Zahn, Ilja Radlgruber, Marius Erik Mayerhoefer, Christoph Grimm, Stephan Polterauer

**Affiliations:** 1 Department of Gynaecology and Gynaecological Oncology, Gynecologic Cancer Unit, Comprehensive Cancer Center, Medical University of Vienna, Vienna, 1090, Austria; 2 Department of Biomedical Imaging and Image-Guided Therapy, Medical University of Vienna, Vienna, Austria, 1090, Austria; The University of Texas MD Anderson Cancer Center, UNITED STATES

## Abstract

**Objective:**

Tumor cachexia is an important prognostic parameter in epithelial ovarian cancer (EOC). Tumor cachexia is characterized by metabolic and inflammatory disturbances. These conditions might be reflected by body composition measurements (BCMs) ascertained by pre-operative computed tomography (CT). Thus, we aimed to identify the prognostically most relevant BCMs assessed by pre-operative CT in EOC patients.

**Methods:**

We evaluated muscle BCMs and well established markers of nutritional and inflammatory status, as well as clinical-pathological parameters in 140 consecutive patients with EOC. Furthermore, a multiplexed inflammatory marker panel of 25 cytokines was used to determine the relationship of BCMs with inflammatory markers and patient’s outcome. All relevant parameters were evaluated in uni- and multivariate survival analysis.

**Results:**

Muscle attenuation (MA)—a well established BCM parameter—is an independent prognostic factor for survival in multivariate analysis (HR 2.25; p = 0.028). Low MA—reflecting a state of cachexia—is also associated with residual tumor after cytoreductive surgery (p = 0.046) and with an unfavorable performance status (p = 0.015). Moreover, MA is associated with Eotaxin and IL-10 out of the 25 cytokine multiplex marker panel in multivariate linear regression analysis (p = 0.021 and p = 0.047, respectively).

**Conclusion:**

MA—ascertained by routine pre-operative CT—is an independent prognostic parameter in EOC patients. Low MA is associated with the inflammatory, as well as the nutritional component of cachexia. Therefore, the clinical value of pre-operative CT could be enhanced by the assessment of MA.

## Introduction

Cancer cachexia is a significant factor contributing to the poor performance status and high mortality rate of cancer patients. Cancer cachexia is a multifactorial and often irreversible syndrome, characterized by metabolic and inflammatory disturbances [1] and accounting for up to 20% of cancer deaths [2]. Thus, evaluating the prognostic value of markers of cachexia and understanding the underlying molecular mechanisms is essential.

In search of markers of cachexia, image analysis of computed tomography (CT) scans has the potential to reveal muscle depletion and to provide potential prognostic imaging biomarkers in cancer patients. In a recent report, Martin *et al* strengthen the possible clinical relevance of body composition measurements (BCMs) obtained from CT scans in cancer patients prior to therapy [3]. Modern image-based estimations of whole body skeletal muscle mass are made via measuring the mean muscle attenuation (MA)–inversely related to muscle fat content—and the skeletal muscle index (SMI) in abdominal cross-sectional CT images at the level of lumbar vertebra 3 (L3) [4] which can also reveal otherwise occult muscle depletion [3].

A progressive and generalized loss of skeletal muscle mass [5] is considered a pathological phenomenon reflecting the wasting process associated with changes in several metabolic pathways in cancer patients [6]. The catabolic process is associated with an alteration in inflammatory cytokines [7] and includes protein synthesis inhibition as well as an increase in protein degradation [8]. Cancer cachexia involves a variety of catabolic proinflammatory cytokines, such as tumor necrosis factor alpha (TNFα) and interleukin (IL)-6, but also anti-inflammatory cytokines such as IL-4 or IL-10 are involved in the pathogenesis of cancer associated metabolic disturbances [9, 10], with a negative impact on prognosis [11].

It has been accepted, that cancer cachexia, associated with an ongoing tumor associated catabolism and systemic inflammatory condition, affects performance status, therapy tolerance, and survival [3, 12, 13]. Due to the lack of typical early symptoms, diagnosis of epithelial ovarian cancer (EOC) is frequently made at advanced disease, which in turn is associated with worsening nutritional and performance outcomes. Unfortunately, an optimal marker of cancer cachexia and an inclusion in clinical routine is still missing, although various studies highlight the importance to consider cachexia associated changes in cancer patients [2, 14].

The present study aimed to investigate the prognostic value of BCMs assessed by pre-operative CT in patients with EOC. The relationship of BCMs with markers of nutritional and inflammatory status will be evaluated using a multiplexed inflammatory marker panel and common clinical-pathological parameters.

## Methods

### Study population

All consecutive patients with primary EOC treated between 2004 and 2012 at the Gynecological Cancer Unit of the Comprehensive Cancer Center at the Medical University of Vienna according to standardized procedures, as previously described in detail [15], were included in the study. Inclusion criteria were: (1) available digitally stored CT scans taken within 30 days pre-operatively for initial pretherapeutic staging and suitable for adequate image analysis; (2) presence of a frozen serum sample collected at time of diagnosis (IRB approval number 366/2003 –Medical University of Vienna; written informed consent was given). In a total of 140 EOC patients, both, preoperative blood samples and digital CT images of adequate quality were available. Clinical and histo-pathological data, laboratory parameters associated with cancer associated cachexia, as well as follow-up data was collected by experienced clinicians and documented prior to therapy. Laboratory analyses were performed as described below. Overall survival (OS) was defined as the time interval between diagnosis and tumor associated death and progression free survival (PFS) as the time between diagnosis and disease progression or death. Overall observation time was the time interval between diagnosis and last contact or date of death. Patients without recurrence, disease progression or non-cancer related death were censored at the time of last follow-up visit. The body mass index (BMI) was calculated as patient weight (kg) / height (m) ^2^, as assessed within 30 days pre-operatively. The BMI was categorized as > 30.0 kg/m^2^, 25.0 to 29.9 kg/m^2^, 18.5 to 24.9 kg/m^2^, and <18.5 kg/m^2^, reflecting obese, overweight, normal weight and underweight patients, respectively.

### Luminex assay description

Multiplexed Luminex-based assays are reliable to investigate the role of selected markers in cancer [16]. We measured circulating levels of cytokine and inflammation markers (IL-1β, IL-1RA, IL-2, IL-2R, IL-4, IL-5, IL-6, IL-7, IL-8, IL-10, IL-12, IL-13, IL-15, IL-17, TNF-α, Granulocyte macrophage colony-stimulating factor (GM-CSF), Macrophage Inflammatory Protein (MIP)-1β, Interferon gamma-induced protein (IP)-10, interferone (IFN)-α, monocyte chemotactic protein (MCP)-1, IFN-γ, monokine induced by gamma-Interferon (MIG), RANTES (regulated on activation, normal T cell expressed and secreted), Eotaxin, and MIP-1α) using a Luminex bead-based commercial assay panel (Cytokine Human Magnetic 25-Plex Panel; Life Technologies), including the most relevant markers linked to ovarian cancer and cancer cachexia.

Blood samples were collected prior to treatment initiation and stored at -80°C. A multiplex panel was used according to the manufacturer`s protocol. The Luminex assay was analyzed using a Bio-Plex 200 array reader (Bio-rad). A quantitative determination of the respective analytes was achieved by comparing the raw data obtained from the patient samples with a standard curve. A total of six cytokines (IL-1β, IL-5, IL-7, IL-15, IL-17, and MIG) had to be excluded because of ≥25% values below detection limit.

### CT Image Analysis

**Fig 1:**
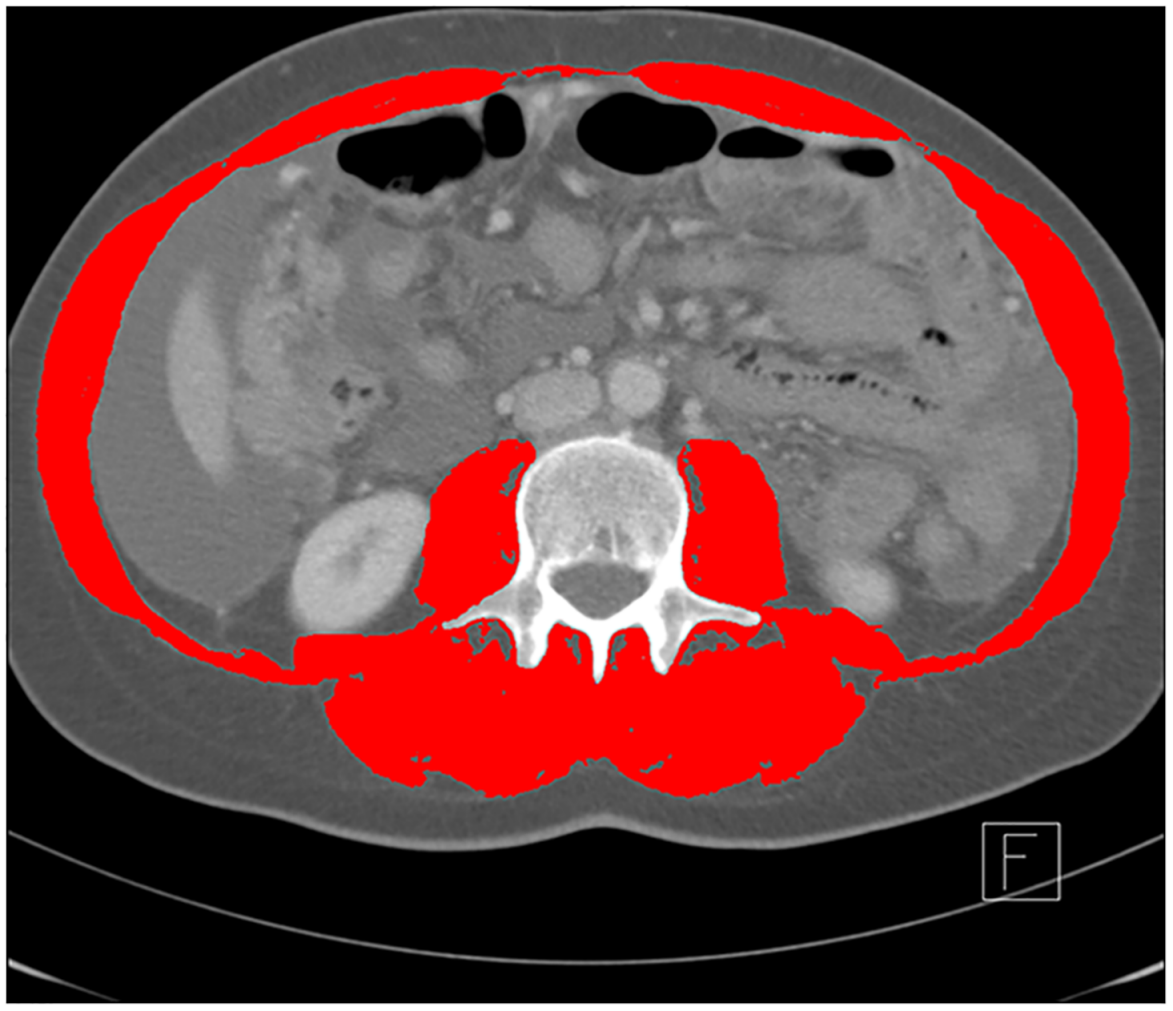
Example of an axial computed tomography (CT) image of the third lumbar vertebra region with the analyzed skeletal muscles highlighted in red.

CT images were acquired using a Siemens Sensation 16 or a Siemens Sensation Cardiac 64 (both Siemens Healthcare, Germany) with 4mm or 3mm slice thickness and 120kVp tube voltage, respectively. CT images were analyzed according to a previously described protocol by using Slice-O-Matic software (v5.0, Tomovision, Montreal, Quebec, Canada) [3]. In CT images, each tissue has a specific radiation attenuation which is measured in absolute numbers in Hounsfield Units (HU) with a range of -1000 HU (air) to +1000 HU (dense bone), e.g. fat ranges from -190 to -30, muscle ranges from -29 to +150 [17]. Thus, the differentiation of fat and muscle is possible and also a quantification of fatty muscle infiltration via calculation of the mean MA. The lower the value of the MA, the higher the fatty infiltration of the muscle. The following muscles were determined and analyzed by two radiologists in consensus on two adjacent axial cross-sectional CT images at the L3 level as estimation from this cross-sectional area and total-body measurements are highly correlated (4): rectus abdominus, abdominal (lateral and oblique), psoas, and paraspinal (quadratus lumborum, erector spinae). Total cross-sectional muscle area (cm^2^) was measured within a range of -29 to +150 HU for the identified muscles on both slices as depicted in Fig 1. Quantified muscle area and calculated mean MA on both slices were averaged for each patient, respectively. The muscle area was normalized for height (meters squared; m^2^) and reported as lumbar skeletal muscle index (SMI) (cm^2^/m^2^).

### Statistical analysis

Statistical analyses were performed using SPSS software version 19 (IBM Corporation, Armonk, New York, United States) and R (R: A Language and Environment for Statistical Computing, R Core Team, 2014, Vienna, Austria). Differences were analyzed using independent *t-* tests for continuous, χ2-tests for categorical variables, and Fisher’s exact test as appropriate. Luminex data were log-transformed to reach normal distribution. Univariate and multivariate analyses for overall survival (OS) and progression free survival (PFS) were conducted using the Cox proportional hazards model; hazard ratios (HRs) and corresponding 95% CIs are shown. To assess also the independent impact of factors not significant in the univariate Cox regression analyses, all factors were included in the multiple models according to suggestions from Harrell [18] and Sun et al. [19]. The optimal cut-off for the MA factor was obtained using the optimal non-linear association of MA with overall survival as determined by the Multivariable Fractional Polynomials method (mfp, R-package) [20]. Correlations and multivariate linear regression analyses with model selection maximizing the Akaike Information Criterion (AIC) were performed to identify independent relationships between BCMs and clinical pathological, laboratory parameters, as well as chemokine and cytokine concentrations. P values of less than 0.05 were considered statistically significant.

## Results

### Patient characteristics

A total of 140 EOC patients undergoing surgical tumor debulking followed by platinum-based chemotherapy between 2004 and 2012 were included. Baseline clinic-pathological characteristics and BCM values are depicted in Table 1. Mean (standard deviation) age at time of diagnosis was 60 (13) years. A total of 103 tumors (73.6%) were of serous histology. In 72.1% (n = 101), tumors were poorly differentiated (high grade), followed by moderately and well differentiated tumors in 19.3% and 8.6% (n = 27 and n = 12, respectively). According to the dualistic model of EOC [21], patients were grouped into high grade serous (type II) *versus* low grade serous and non-serous tumors (type I; Table 1). Complete cytoreductive surgery with no evidence of residual tumor could be achieved in 54.3% (n = 76). Out of the 64 patients with macroscopically visual residual tumor, the tumor load was < 5mm in 15 patients (10.7%) and >2cm in 33 patients (23.6%). A total of 56 patients (40%) died of disease and 93 patients (66%) experienced tumor recurrence. Median (interquartile range) follow up time was 56 (33–73) months.

**Table 1:** Patient characteristics.

Characteristics	n (%)
**ECOG**	
0	120 (85.7)
1	15 (10.7)
2	3 (2.1)
3	2 (1.4)
**FIGO**	
I	19 (13.6)
II	10 (7.1)
III	86 (61.4)
IV	25 (17.9)
**Type**	
II (high grade serous)	98 (70.0)
I (low grade serous, non serous)	42 (30.0)
**Residual tumor**	
no	76 (54.3)
> 0 cm	64 (45.7)
**BMI** *	
<18.5	4 (2.9)
18.5–24.9	73 (52.1)
25.0–29.9	33 (23.6)
>30.0	21 (15.0)
**Muscle attenuation**	
< 39 HU	49 (35)
> 39 HU	91 (65)
**Skeletal muscle index** **	
< 41 (cm^2^/m^2^)	39 (28.9)
> 41 (cm^2^/m^2^)	96 (71.1)

n = 140;

* 9 missing;

** 5 missing

### Body composition measurements

**Fig 2:**
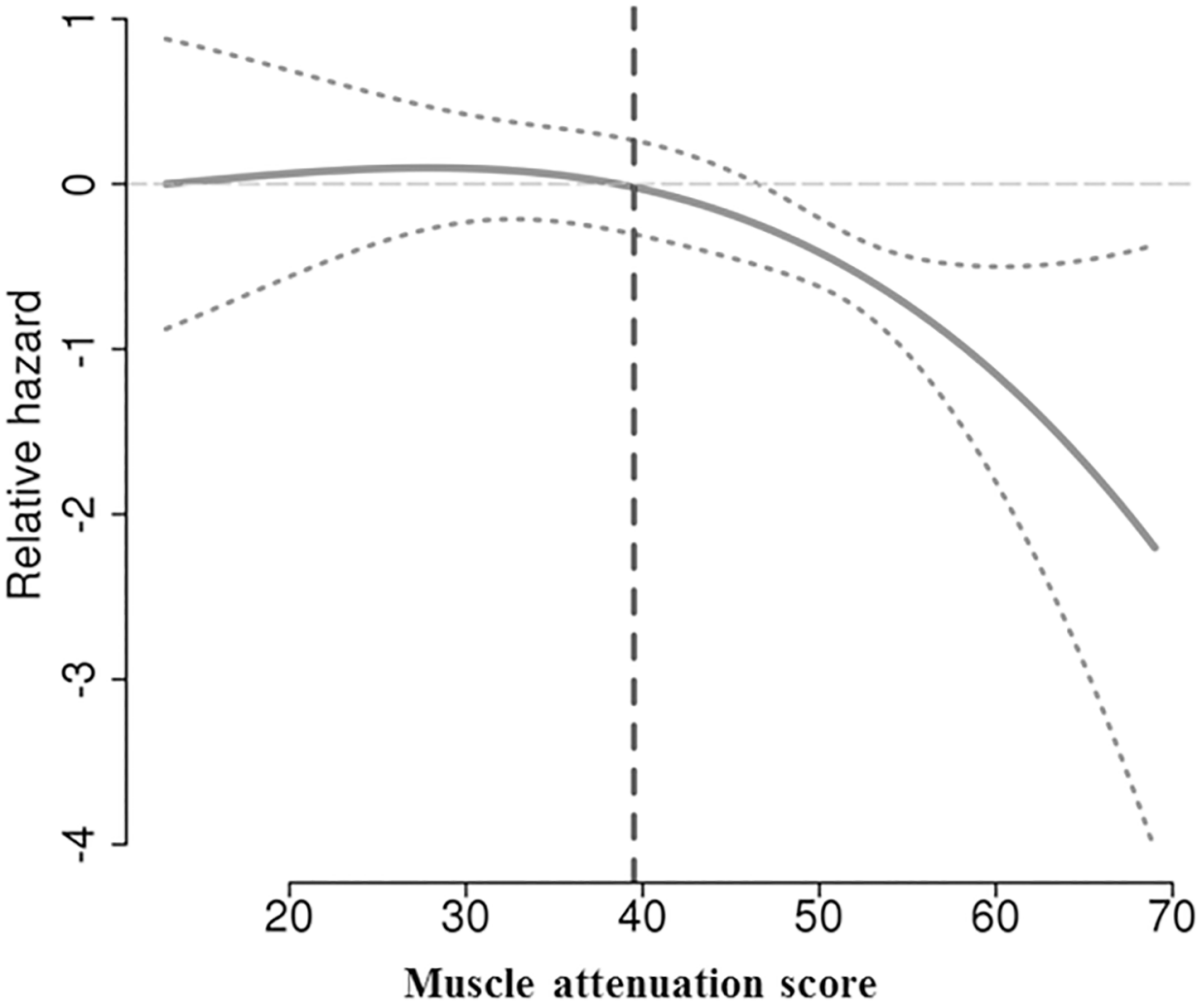
Relative risk of death on the basis of the muscle attenuation (MA) score; optimal cutoff set at 39 as inferred by the Multivariable Fractional Polynomials (mfp) R-package using the optimal non-linear association (MA/100)^2^+(MA/100)^3^ with overall survival.

A wide variation of the BCMs could be observed in our study population. The mean BMI was 24.9 (4.8) kg/m^2^. At time of diagnosis, only 2.9% (n = 4) of patients were classified as underweight. On the other hand, a total of 38.6% (n = 54) was classified as obese or overweight. The mean lumbar total muscle cross-sectional area was 120.3 cm^2^ (18.6). The mean SMI was 44.9 cm^2^/m^2^ (7.4). For statistical analyses, the cut-off for high and low SMI was set at 41 cm^2^/m^2^, previously reported as significantly associated with survival in female cancer patients. This threshold was defined according to sex and BMI revealing the cutoff <41 (cm2/m2)–stable among all four BMI groups. [3]. Thecut-off for high and low MA was newly defined and set at 39 HU according to the relative risk of death related to the MA values (Fig 2). We decided to define the optimal MA threshold for this population, as a previously reported threshold differed according to the patient’s BMI [3]. The majority of patients with low MA (57.1%) had residual tumor after cytoreduction, whereas optimal cytoreduction could be archieved in 60.4% of patients with high MA (p = 0.046; Table 2). Interestingly, a low BMI did not correspond to a low MA. In our cohort, not only normal weight, but also underweight patients showed a significantly higher MA compared to overweight and obese EOC patients (Table 2). In contrast, the SMI was significantly lower in underweight compared to obese women (p<0.001). Decreasing MA did correlate with increasing age (Spearman-Rho coefficient: r -0,537; p < 0.001;). The mean MA was significantly lower in fully active patients (Eastern Cooperative Oncology Group performance status (ECOG) 0: mean MA 45 HU, SD 12 HU) compared to physically restricted patients (ECOG 1–3: mean MA 38 HU, SD 9 HU; p = 0.020) whereas no significant difference could be observed in the mean BMI related to the ECOG performance status (ECOG 0: mean BMI 25.04, SD 5.03; ECOG 1–3: mean BMI 24.36, SD 3.75; p = 0.565).

**Table 2:** Patient characteristics stratified into low and high muscle attenuation (MA) scores.

Characteristics	Low MA (n = 49) n (%)	High MA (n = 91) n (%)	p
**ECOG performance status**			**0.015** *
0 (n = 120)	37 (75.5)	83 (91.2)	
1 (n = 15)	8 (16.3)	7 (7.7)	
2 (n = 3)	3 (6.1)	0 (0.0)	
3 (n = 2)	1 (2.0)	1 (1.1)	
**FIGO stage**			0.501*
I (n = 19)	4 (8.2)	15 (16.5)	
II (n = 10)	3 (6.1)	7 (7.7)	
III (n = 86)	34 (96.4)	53 (58.2)	
IV (n = 25)	8 (16.3)	16 (17.6)	
**Type**			0.153**
II (high grade serous, n = 42)	38 (77.6)	60 (65.9)	
I (low grade serous, non serous, n = 98)	11 (22.4)	31 (34.1)	
**Residual tumor**			**0.046** **
No (n = 76)	21 (42.9)	55 (60.4)	
> 0cm (n = 64)	28 (57.1)	36 (39.6)	
**BMI** (9 missing)			**<0.001** *
<18.5 (n = 4)	0 (0.0)	4 (4.8)	
18.5–24.9 (n = 73)	17 (36.2)	56 (66.7)	
25.0–29.9 (n = 33)	16 (34.0)	17 (20.2)	
>30.0 (n = 21)	14 (29.8)	7 (8.3)	
**skeletal muscle index** (5 missing)			
< 41 (cm^2^/m^2^; n = 39)	12 (25.0)	27 (31.0)	0.553**
> 41 (cm^2^/m^2^; n = 96)	36 (75.0)	60 (69.0)	

n = 140;

*Fisher exact,

** Students’ t-test

### Impact of clinical-pathological factors and body composition measurements on overall survival

**Fig 3:**
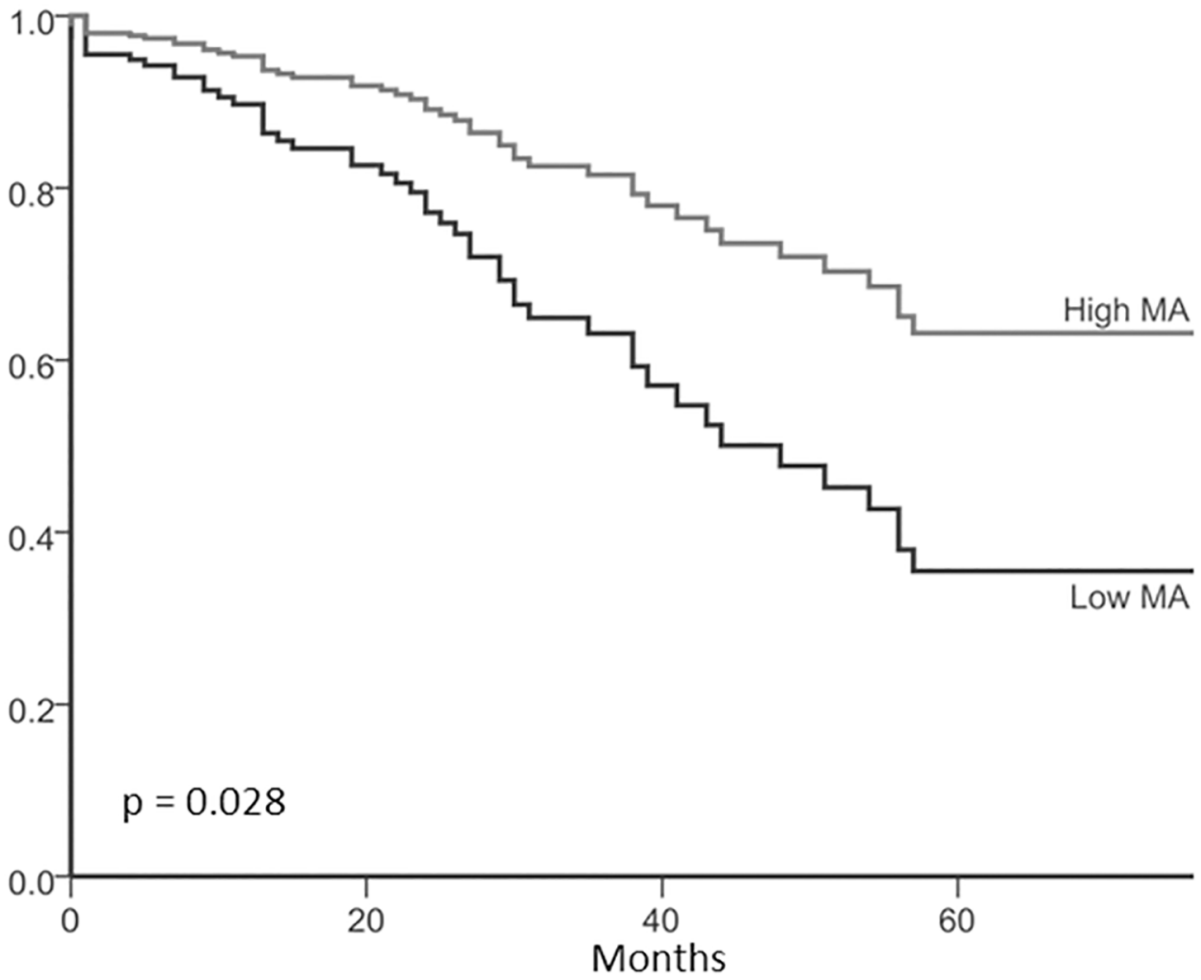
Overall survival stratified by muscle attenuation (MA). The present Kaplan-Meier-Curve has been corrected for the relevant clinic pathologic and anthropometric parameters.

Table 3 shows results of univariate and multivariate survival analyses investigating well known clinical-pathological parameters, such as age, performance status, the International Federation of Gynecology and Obstetrics (FIGO) stage, type, residual tumor, as well as BMI and CT BCMs. Based on a multiple Cox-regression analysis, the final model revealed MA, together with age, FIGO stage, and suboptimal cytoreductive surgery (residual macroscopic tumor) as independently associated with OS. Using the BMI dependent cutoffs for MA as described by Martin et al [3] revealed a significant impact on OS in univariate (HR 2.35, p = 0.002) but not in multiple analysis (HR 1.67, p = 0.138; S1 Table). The impact of MA, corrected for the relevant clinical-pathological and BCM parameters, on prognostication of OS is shown in Fig 3 as estimated survival curves.

**Table 3:** Survival analysis of known clinical-pathological parameters together with body composition measurements (BMI, SMI, and MA).

	Progression Free Survival				Overall Survival			
	Univariate^1^		Multivariate^2^		Univariate^1^		Multivariate^2^	
	HR (95%CI)	*P*	HR (95%CI)	*P*	HR (95%CI)	*P*	HR (95%CI)	*P*
**Age** (decades)	**1.32 (1.12–1.55)**	**0.001**	**1.27 (1.04–1.56)**	**0.018**	**1.69 (1.35–2.13)**	**<0.001**	**1.55 (1.18–2.03)**	**0.002**
**ECOG status** (0 *vs* 1 *vs* 2 *vs* 3)	1.23 (0.88–1.72)	0.220	1.20 (0.81–1.79)	0.360	**1.61 (1.10–2.35)**	**0.014**	1.51 (0.97–1.35)	0.068
**FIGO stage** (I *vs* II *vs* III *vs* IV)	**2.44 (1.82–3.27)**	**<0.001**	**2.16 (0.75–2.38)**	**0.000**	**2.63 (1.77–3.90)**	**<0.001**	**2.54 (1.56–4.15)**	**0.000**
**Type** (type1 *vs*. type2)	**2.13 (1.30–3.51)**	**0.003**	1.34 (1.12–2.92)	0.322	**1.96 (1.03–3.74)**	**0.041**	0.95 (0.45–1.99)	0.897
**Residual tumor** (no *vs*. yes)	**3.35 (2.19–5.14)**	**<0.001**	**1.81 (0.93–1.06)**	**0.015**	**3.43 (1.96–5.99)**	**<0.001**	**1.84 (1.02–3.34)**	**0.045**
**BMI**	0.98 (0.94–1.03)	0.471	0.99 (0.76–2.26)	0.926	0.96 (0.90–1.02)	0.170	0.92 (0.84–1.01)	0.085
**SMI** (</> 41cm^2^/m^2^)	1.13 (0.71–1.81)	0.605	1.31 (0.76–2.26)	0.336	0.92 (0.50–1.68)	0.786	1.23 (0.61–2.48)	0.565
**MA** (high *vs* low)	**1.54 (1.01–2.34)**	**0.046**	1.22 (0.69–2.17)	0.500	**2.41 (0.24–0.70)**	**0.001**	**2.25 (1.09–4.65)**	**0.028**

(n = 140;

^1^ Log rank test;

^2^ multivariate Cox-regression analysis,

HR = Hazard Ratio, 95%CI = 95% Confidence Interval, ECOG = Eastern Cooperative Oncology Grouppe, FIGO = BMI = body mass index, SMI = skeletal muscle index, MA = muscle attenuation)

### Correlation of inflammatory parameters with MA

To understand the biological factors associated with low MA, basic laboratory parameters available in clinical routine and know to be associated with low nutrition status and tumor associated inflammation—namely albumin, total protein, leukocytes, and C-reactive protein (CRP)—were correlated with MA. The correlation was strongest for albumin, with a significantly positive correlation of higher albumin levels with a higher median MA (p = 0.013), Likewise, higher protein levels were related to higher MA, though not reaching statistical significance (p = 0.064). The other parameters showed no statistically significant correlation with MA (data not shown).

### Correlation of cytokines with MA

Numerous cytokines seem to play a role in the metabolic changes associated with wasting and cancer cachexia. To determine the relationship between inflammatory processes and low MA, the serum levels of a set of 25 cytokines were evaluated, including the pro-cachectic cytokines TNFα, IL-6, IL-1 and IFN-y as well as anti-cachectic cytokines such as IL-10, IL-4, IL-13, and IL-1RA [22] and important markers previously analyzed in ovarian cancer [23]. Multivariate linear regression analysis revealed that Eotaxin and IL-10 were independent predictors of MA. IL-13, IL-2R, and IL-4 remained as correcting parameters in the final model. Results of the analyses are depicted in Table 4.

**Table 4:** Linear regression analysis of the relationship between plasma-cytokines and MA. The univariate analysis is only shown for the 5 parameters remaining in the final multivariate model; 95%CI = 95% Confidence Interval.

	MA			
	Univariate		Multivariate	
	Coeff (95%CI)	*P*	Coeff (95%CI)	*P*
**Eotaxin**	**-0.97 (-3.09; 1.16)**	**0.377**	**-4.00 (-7.31; -0.68)**	**0.021**
**IL-10**	-0.70 (-1.72; 0.31)	0.180	**-1.17 (-2.29; -0.04)**	**0.047**
IL-13	1.65 (-0.09; 3.39)	0.068	1.62 (-0.15; 3.39)	0.077
IL-2R	**1.93 (0.09; 3.77)**	**0.043**	1.73 (-0.63; 4.08)	0.156
IL-4	0.18 (0.77; 1.14)	0.703	1.29 (-0.54; 3.14)	0.172

## Discussion

The importance of cancer cachexia and the nutritional state in ovarian cancer prognosis has been recognized. This study demonstrates that MA, which seems to be associated with cancer cachexia, is an independent prognostic parameter easily ascertained by routine pre-operative CT in EOC patients.

Trying to measure cancer related metabolic and inflammatory changes, an increased infiltration of skeletal muscle by inter- and intramuscular fat can be quantified by analysis of BCMs such as MA in CT scans [4, 24]. Still, little is known about the prognostic relevance of BCMs determined by CT scan analysis, especially in EOC, although it is well known that malnutrition and muscle depletion affect surgical outcome and postoperative morbidity and mortality in cancer patients [25].

We found a significantly reduced overall survival in EOC patients with low MA—information of importance in the course of individualized treatment planning. A reduced SMI showed no significant impact on survival in EOC. Additionally, previous studies showed that the value of adipose tissue measurement was lacking clinical significance [26]. A significant association of low MA with reduced overall or progression-free survival has been described in patients with tumors of the gastrointestinal tract, the lung, in renal cell carcinoma, and in melanoma patients [3, 26, 27].

Despite significant improvements in treatment and understanding of metabolic and inflammatory pathways, recent studies reported that malnutrition still continues to be a significant challenge in ovarian cancer management [3, 26, 27].

One could hypothesize, that the BMI of EOC patients might correspond to the measured MA. However, in our patient population not only normal weight, but also underweight patients showed a significantly higher MA compared to overweight and obese EOC patients. This underlines, that BMI alone is a relatively inaccurate measurement of muscle composition and cancer cachexia in EOC. This is comparable to findings in patients with lung- or GI cancer [3]. Furthermore, we observed that MA was significantly lower in physically restricted patients, whereas the patients’ BMI was not associated with the ECOG performance status.

MA was also significantly associated with the presence of residual tumor after cytoreduction. The presence of any post-operative residual tumor is a powerful factor with a tremendous impact on OS. [28]. Thus, CT scan analysis might help to identify those patients, in whom optimal cytoreduction is unlikely to be achieved.

In the present study we also investigated the biochemical processes corresponding to this condition.

We found that patients with low MA presented in a worse nutritional and systemic inflammatory status reflected among others by lower protein and albumin levels. Pre-operative serum albumin is an accepted marker of poor nutrition in EOC [29]. In solid tumors, albumin seems to accumulate in the tumor microenvironment, and cancer cells seem to have the ability to utilize albumin as source of energy [30, 31]. The rate of albumin synthesis is not only influenced by nutrition but also inflammation, reflected in the observation that albumin is a negative acute phase protein. Considering inflammation, we tried to understand the biochemical processes related to a low MA by measuringthe concentration of a multiplexed inflammatory marker panel of 25 cytokines, revealing Eotaxin-1 together with the anti-inflammatory cytokine IL-10 as significantly associated with MA.

Cancer is highly connected to the patient’s immune response and inflammation plays an important role in muscle function. The imbalance between pro- and anti-tumor immunological profiles, as reflected by cytokines, increases with tumor progression. The potent anti-inflammatory factor IL-10 induces systemic tumor-specific immunity and plays an important role in the control of tumor-promoting inflammation [32, 33]. The IL-10 network is probably the most relevant link between cancer and inflammation [34]. In EOC, an impaired anti-tumor immune response seems to be associated with increased IL-10 levels [35], also reflected in higher levels in high grade compared to low grade tumors [36]. Similar to IL-10, Eotaxin-1 has been mainly studied in inflammatory disorders [37]. In EOC, cancer-dependent changes in Eotaxin-1 levels were demonstrated [38]. In gastric cancer, Eotaxin-1 levels were even proposed as biomarker for early diagnosis [39].

The measurement of MA on routine pre-operative CT is easy to perform, reproducible, ready available, and cheap. Aubrey et al. already mentioned a possible merit by including the quantification of attenuation in the repertoire of radiologists [17]. Our results showed impaired overall survival and unfavorable surgical outcome in patients with EOC and low MA in a multivariate survival analysis, highlighting the importance of this easy-accessible marker.

Limitations of the study include its retrospective design, resulting in a lack of information on important co-variates such as the amount of tumor load and the amount of ascites. Furthermore, research would benefit from standardized diagnostic criteria for low MA that could be used for individualized care—such as nutritional management—of patients with EOC.

## Conclusion

This study demonstrates that without much effort, the measurement of MA in pre-operative CT scans of EOC patients could be utilized to become aware of the systemic muscle condition and the related predictive and prognostic implications that should be addressed in course of individual treatment planning.

## Supporting Information

S1 Table Survival analysis. Known clinical-pathological parameters together with body composition measurements (BMI, SMI, and MA as categorized by Martin et al in a BMI dependent way (3); n = 140).
(DOCX)

## References

[1] Faber J, Vos AP, Kegler D, Argiles J, Laviano A, Garssen J, et al Impaired immune function: an early marker for cancer cachexia. Oncology reports. 2009;22(6):1403–6. . 19885593 10.3892/or_00000581

[2] Argiles JM, Busquets S, Stemmler B, Lopez-Soriano FJ. Cancer cachexia: understanding the molecular basis. Nature reviews Cancer. 2014;14(11):754–62. 10.1038/nrc3829 . 25291291

[3] Martin L, Birdsell L, Macdonald N, Reiman T, Clandinin MT, McCargar LJ, et al Cancer cachexia in the age of obesity: skeletal muscle depletion is a powerful prognostic factor, independent of body mass index. J Clin Oncol. 2013;31(12):1539–47. Epub 2013/03/27. 10.1200/JCO.2012.45.2722 [pii]. . 23530101

[4] Shen W, Punyanitya M, Wang Z, Gallagher D, St-Onge MP, Albu J, et al Total body skeletal muscle and adipose tissue volumes: estimation from a single abdominal cross-sectional image. J Appl Physiol (1985). 2004;97(6):2333–8. Epub 2004/08/18. 10.1152/japplphysiol.00744.2004 [pii]. . 15310748

[5] Cruz-Jentoft AJ, Baeyens JP, Bauer JM, Boirie Y, Cederholm T, Landi F, et al Sarcopenia: European consensus on definition and diagnosis: Report of the European Working Group on Sarcopenia in Older People. Age and ageing. 2010;39(4):412–23. 10.1093/ageing/afq034 20392703 PMC2886201

[6] Delmonico MJ, Harris TB, Lee JS, Visser M, Nevitt M, Kritchevsky SB, et al Alternative definitions of sarcopenia, lower extremity performance, and functional impairment with aging in older men and women. J Am Geriatr Soc. 2007;55(5):769–74. Epub 2007/05/12. JGS1140 [pii] 10.1111/j.1532-5415.2007.01140.x . 17493199

[7] McNamara MJ, Alexander HR, Norton JA. Cytokines and their role in the pathophysiology of cancer cachexia. JPEN Journal of parenteral and enteral nutrition. 1992;16(6 Suppl):50S–5S. . 1287224 10.1177/014860719201600603

[8] Suzuki H, Asakawa A, Amitani H, Nakamura N, Inui A. Cancer cachexia—pathophysiology and management. J Gastroenterol. 2013;48(5):574–94. Epub 2013/03/21. 10.1007/s00535-013-0787-0 23512346 PMC3698426

[9] Argiles JM, Busquets S, Lopez-Soriano FJ. Cytokines in the pathogenesis of cancer cachexia. Curr Opin Clin Nutr Metab Care. 2003;6(4):401–6. . 12806213 10.1097/01.mco.0000078983.18774.cc

[10] Argiles JM, Busquets S, Garcia-Martinez C, Lopez-Soriano FJ. Mediators involved in the cancer anorexia-cachexia syndrome: past, present, and future. Nutrition. 2005;21(9):977–85. 10.1016/j.nut.2005.02.003 . 16043325

[11] Tempfer C, Zeisler H, Sliutz G, Haeusler G, Hanzal E, Kainz C. Serum evaluation of interleukin 6 in ovarian cancer patients. Gynecol Oncol. 1997;66(1):27–30. 10.1006/gyno.1997.4726 . 9234916

[12] Bachmann J, Heiligensetzer M, Krakowski-Roosen H, Buchler MW, Friess H, Martignoni ME. Cachexia worsens prognosis in patients with resectable pancreatic cancer. Journal of gastrointestinal surgery: official journal of the Society for Surgery of the Alimentary Tract. 2008;12(7):1193–201. 10.1007/s11605-008-0505-z . 18347879

[13] Deans C, Wigmore SJ. Systemic inflammation, cachexia and prognosis in patients with cancer. Curr Opin Clin Nutr Metab Care. 2005;8(3):265–9. Epub 2005/04/06. 00075197-200505000-00005 [pii]. . 15809528 10.1097/01.mco.0000165004.93707.88

[14] Fearon K, Strasser F, Anker SD, Bosaeus I, Bruera E, Fainsinger RL, et al Definition and classification of cancer cachexia: an international consensus. Lancet Oncol. 2011;12(5):489–95. Epub 2011/02/08. 10.1016/S1470-2045(10)70218-7 [pii]. . 21296615

[15] Aust S, Horak P, Pils D, Pils S, Grimm C, Horvat R, et al The prognostic value of estrogen receptor beta and proline-, glutamic acid- and leucine-rich protein 1 (PELP1) expression in ovarian cancer. BMC cancer. 2013;13:115 10.1186/1471-2407-13-115 23497172 PMC3605348

[16] Weissman AD, Broussolle EP, London ED. In vivo binding of [3H]d-N-allylnormetazocine and [3H]haloperidol to sigma receptors in the mouse brain. Journal of chemical neuroanatomy. 1990;3(5):347–54. . 2171560

[17] Aubrey J, Esfandiari N, Baracos VE, Buteau FA, Frenette J, Putman CT, et al Measurement of skeletal muscle radiation attenuation and basis of its biological variation. Acta physiologica. 2014;210(3):489–97. 10.1111/apha.12224 . 24393306 PMC4309522

[18] Harrell FE. Regression modeling strategies: with applications to linear models, logistic regression, and survival analysis. New York: Springer; 2001 xxii, 568 p. p.

[19] Sun GW, Shook TL, Kay GL. Inappropriate use of bivariable analysis to screen risk factors for use in multivariable analysis. Journal of clinical epidemiology. 1996;49(8):907–16. . 8699212 10.1016/0895-4356(96)00025-x

[20] Royston P, Altman DG. Regression using fractional polynomials of continuous covariates: parsimonious parametric modelling. Appl Statist. 1994;43(3):429–67.

[21] Kurman RJ, Shih Ie M. Pathogenesis of ovarian cancer: lessons from morphology and molecular biology and their clinical implications. International journal of gynecological pathology: official journal of the International Society of Gynecological Pathologists. 2008;27(2):151–60. 18317228 10.1097/PGP.0b013e318161e4f5 PMC2794425

[22] Argiles JM, Moore-Carrasco R, Fuster G, Busquets S, Lopez-Soriano FJ. Cancer cachexia: the molecular mechanisms. The international journal of biochemistry & cell biology. 2003;35(4):405–9. . 12565701 10.1016/s1357-2725(02)00251-0

[23] Block MS, Maurer MJ, Goergen K, Kalli KR, Erskine CL, Behrens MD, et al Plasma immune analytes in patients with epithelial ovarian cancer. Cytokine. 2015;73(1):108–13. 10.1016/j.cyto.2015.01.035 . 25743245 PMC4380743

[24] Goodpaster BH, Thaete FL, Kelley DE. Composition of skeletal muscle evaluated with computed tomography. Ann N Y Acad Sci. 2000;904:18–24. . 10865705 10.1111/j.1749-6632.2000.tb06416.x

[25] Prado CM, Lieffers JR, McCargar LJ, Reiman T, Sawyer MB, Martin L, et al Prevalence and clinical implications of sarcopenic obesity in patients with solid tumours of the respiratory and gastrointestinal tracts: a population-based study. Lancet Oncol. 2008;9(7):629–35. 10.1016/S1470-2045(08)70153-0 . 18539529

[26] Antoun S, Lanoy E, Iacovelli R, Albiges-Sauvin L, Loriot Y, Merad-Taoufik M, et al Skeletal muscle density predicts prognosis in patients with metastatic renal cell carcinoma treated with targeted therapies. Cancer. 2013;119(18):3377–84. 10.1002/cncr.28218 . 23801109

[27] Sabel MS, Lee J, Cai S, Englesbe MJ, Holcombe S, Wang S. Sarcopenia as a prognostic factor among patients with stage III melanoma. Annals of surgical oncology. 2011;18(13):3579–85. 10.1245/s10434-011-1976-9 . 21822551

[28] Nick AM, Coleman RL, Ramirez PT, Sood AK. A framework for a personalized surgical approach to ovarian cancer. Nature reviews Clinical oncology. 2015 10.1038/nrclinonc.2015.26 . 25707631 PMC4528308

[29] Asher V, Lee J, Bali A. Preoperative serum albumin is an independent prognostic predictor of survival in ovarian cancer. Medical oncology. 2012;29(3):2005–9. 10.1007/s12032-011-0019-5 . 21735143

[30] Merlot AM, Kalinowski DS, Richardson DR. Unraveling the mysteries of serum albumin-more than just a serum protein. Frontiers in physiology. 2014;5:299 10.3389/fphys.2014.00299 25161624 PMC4129365

[31] Stehle G, Sinn H, Wunder A, Schrenk HH, Stewart JC, Hartung G, et al Plasma protein (albumin) catabolism by the tumor itself—implications for tumor metabolism and the genesis of cachexia. Critical reviews in oncology/hematology. 1997;26(2):77–100. . 9298326 10.1016/s1040-8428(97)00015-2

[32] Neven B, Mamessier E, Bruneau J, Kaltenbach S, Kotlarz D, Suarez F, et al A Mendelian predisposition to B-cell lymphoma caused by IL-10R deficiency. Blood. 2013;122(23):3713–22. 10.1182/blood-2013-06-508267 . 24089328

[33] Berg DJ, Davidson N, Kuhn R, Muller W, Menon S, Holland G, et al Enterocolitis and colon cancer in interleukin-10-deficient mice are associated with aberrant cytokine production and CD4(+) TH1-like responses. The Journal of clinical investigation. 1996;98(4):1010–20. 10.1172/JCI118861 8770874 PMC507517

[34] Acuner-Ozbabacan ES, Engin BH, Guven-Maiorov E, Kuzu G, Muratcioglu S, Baspinar A, et al The structural network of Interleukin-10 and its implications in inflammation and cancer. BMC genomics. 2014;15 Suppl 4:S2 25056661 10.1186/1471-2164-15-S4-S2 PMC4083408

[35] Santin AD, Bellone S, Ravaggi A, Roman J, Smith CV, Pecorelli S, et al Increased levels of interleukin-10 and transforming growth factor-beta in the plasma and ascitic fluid of patients with advanced ovarian cancer. BJOG: an international journal of obstetrics and gynaecology. 2001;108(8):804–8. . 11510703 10.1111/j.1471-0528.2001.00206.x

[36] Tsai-Turton M, Santillan A, Lu D, Bristow RE, Chan KC, Shih Ie M, et al p53 autoantibodies, cytokine levels and ovarian carcinogenesis. Gynecol Oncol. 2009;114(1):12–7. 10.1016/j.ygyno.2009.03.028 19398128 PMC2694938

[37] Rankin SM, Conroy DM, Williams TJ. Eotaxin and eosinophil recruitment: implications for human disease. Molecular medicine today. 2000;6(1):20–7. . 10637571 10.1016/s1357-4310(99)01635-4

[38] Levina V, Nolen BM, Marrangoni AM, Cheng P, Marks JR, Szczepanski MJ, et al Role of eotaxin-1 signaling in ovarian cancer. Clinical cancer research: an official journal of the American Association for Cancer Research. 2009;15(8):2647–56. 10.1158/1078-0432.CCR-08-2024 19351767 PMC2669845

[39] Koc U, Cetinkaya E, Bostanci EB, Kemik AS, Tez M, Gomceli I, et al Diagnostic significance of serum eotaxin-1 level in gastric cancer patients. Disease markers. 2013;35(5):363–7. 10.1155/2013/274515 24223454 PMC3810232

