# Supplementary material for: Skeletal Muscle Depletion and Markers for Cancer Cachexia Are Strong Prognostic Factors in Epithelial Ovarian Cancer
Source: PLoS One. 2015 Oct 12;10(10):e0140403. doi: 10.1371/journal.pone.0140403 (PMC4601693; doi:10.1371/journal.pone.0140403)
Supplement: S1 Table — Known clinical-pathological parameters together with body composition measurements (BMI, SMI, and MA as categorized by Martin et al in a BMI dependent way (3); n = 140). (DOCX) [file pone.0140403.s001.docx]

**Supporting information**

|  |  | **Overall Survival** | | | |
| --- | --- | --- | --- | --- | --- |
|  |  | **Univariate^1^** | | **Multivariate^2^** | |
|  |  | **HR (95%CI)** | ***P*** | **HR (95%CI)** | ***P*** |
| **Age**  (decades) |  | **1.69 (1.35-2.13)** | **<0.001** | **1.71 (1.29-2.28)** | **<0.001** |
| **ECOG status**  (0 *vs* 1 *vs* 2 *vs* 3) |  | **1.61 (1.10-2.35)** | **0.014** | **1.82 (1.12-2.97)** | **0.016** |
| **FIGO stage**  (I *vs* II *vs* III *vs* IV) |  | **2.63 (1.77-3.90)** | **<0.001** | **1.78 (1.31-2.43)** | **<0.001** |
| **Type**  (type1 *vs*. type2) |  | **1.96 (1.03-3.74)** | **0.041** | 0.82 (0l40-1.68) | 0.593 |
| **Residual tumor**  (no *vs*. yes) |  | **3.43 (1.96-5.99)** | **<0.001** | 1.41 (0.77-2.67) | 0.264 |
| **BMI** |  | 0.96 (0.90-1.02) | 0.170 | 0.93 (0.85-1.02) | 0.121 |
| **SMI**  (</> 41cm²/m²) |  | 0.92 (0.50-1.68) | 0.786 | 1.43 (0.72-2.82) | 0.310 |
| **MA**  (high *vs* low) |  | **2.41 (0.24-0.70)** | **0.001** | 1.44 (0.73-2.87) | 0.297 |

**S1 Table. Survival analysis.** Known clinical-pathological parameters together with body composition measurements (BMI, SMI, and MA as categorized by Martin et al in a BMI dependent way (3); n=140)
